# The dialectics of trauma and political conscientization: A psychosocial study of activism for supporting sexual and gender minoritized communities in Brazil

**DOI:** 10.1002/jts.70054

**Published:** 2026-02-25

**Authors:** Gab C. Siqueira, Luisa F. Velásquez Vallejo, Antonio Euzebios Filho

**Affiliations:** ^1^ Behavioural and Social Sciences University of Groningen Groningen the Netherlands; ^2^ Institute of Psychology University of São Paulo São Paulo Brazil; ^3^ Faculty of Arts University of Groningen Groningen the Netherlands

## Abstract

This qualitative study examined the dialectical association between psychosocial trauma and political conscientization in the lives of activists advocating for persons with marginalized sexual orientations and gender identities (2SLGBTQIA+) in São José dos Campos, Brazil. Drawing on participatory research and grounded in liberation psychology and intersectionality, the study involved collaboration with 10 activists through participation in advocacy activities, semistructured interviews, and collective reflection. Our reflexive thematic analysis indicated four main themes: (a) *addressing psychosocial harm and trauma in the realm of sexual orientation and gender identity*, highlighting how activists navigate the effects of historical violence and activist work and employ strategies of self‐ and collective care; (b) *decoding and confronting intersecting power structures*, capturing how activists interpret and resist institutional and everyday oppression; (c) *reclaiming and preserving collective memory*, describing activists’ efforts to resignify and document 2SLGBTQIA+ histories through art, research, and the transformation of cultural traditions; and (d) *cultivating citizenship and political consciousness*, reflecting the pursuit of rights and recognition by pressuring state institutions and creating autonomous spaces for community support. These interwoven themes illustrate how activism is lived as a dialectical process, both a site of conflict exposure and a space for resignifying suffering, fostering critical awareness, and enacting collective agency. The analysis highlights that responses to psychosocial trauma and resistance are deeply interconnected and continually negotiated through conscientization and collective engagement. Findings indicate that social movements, health professionals, and researchers should support intersectional, community‐led initiatives centering psychosocial care, critical reflection, and the dialectical reinterpretation of collective memory.

Two‐spirit, lesbian, gay, bisexual, trans, queer, intersex, asexual, and other persons with marginalized sexual orientations and gender identities (2SLGBTQIA+) worldwide encounter frequent, sequential, and severe experiences of oppression and discrimination based on their sexual orientation and gender identity or expression (SOGIE), and these experiences are often interpreted as traumatic events (Carneiro et al., 2024; Frost et al., 2019). For individuals who also contend with intersecting systems of oppression, such as racism, ableism, poverty, or HIV‐related stigma, these harms are compounded, producing unique and intensified forms of psychosocial trauma (PST; Obenauf et al., 2024; Pepin‐Neff & Wynters, 2020; Szymanski et al., 2023). From a liberation psychology (LP) perspective, PST is understood as the embodiment of dehumanizing social relations (Martín‐Baró, 1989) produced through systemic oppression and political violence, with profound consequences for mental and physical health, as well as for collective life, including the erosion of social trust, communal bonds, and the capacity for collective action (Euzébios Filho, 2023). Although PST operates at the collective level, it is lived in differentiated ways, as intersectional power relations shape how individuals are positioned within structures of marginalization and access to resources (Collins, 2020). Accordingly, LP conceptualizes PST beyond individual symptoms or pathology, situating it as a historical, political, and relational phenomenon.

LP, developed in Latin America in the 1980s as a branch of critical social psychology (Martín‐Baró, 1989), is a praxis (i.e., theory and practice) that centers the agency and struggles of oppressed peoples. It situates psychosocial suffering within economic, political, cultural, and historical contexts and addresses PST by fostering political consciousness, historical memory, and resistance (Euzébios Filho, 2023). Another central concept in LP is *conscientization*, a dialectical psychosocial phenomenon through which people cultivate critical consciousness of their political reality while simultaneously transforming it by interrogating power relations and systems of oppression (Freire, 1970). Within this framework, psychosocial phenomena are understood as dialectical—that is, they manifest in relation to one another through contradiction, tension, and transformation. From this perspective, PST and conscientization are dialectical processes, with one or the other becoming predominant depending on historical, material, and social conditions.

LP has guided research on PST in diverse contexts, including civil wars (Martín‐Baró, 1989), authoritarian violence, Black movements and state‐forced displacement (Matsumoto et al., 2024), natural disasters (Oliveira & Pontes, 2021), and violence against Indigenous peoples (Gonçalves, 2017). In these struggles, conscientization has consistently emerged as a central process of social change. However, this perspective has rarely been extended to examine resistance among 2SLGBTQIA+ populations (Singh et al., 2023). Our study addressed this challenge by examining how PST unfolds in 2SLGBTQIA+ activist contexts, where resistance is dialectically enacted through collective practices.

To advance this analysis, we drew on intersectionality as a “critical inquiry and praxis” (Collins, 2020, p. 123) to examine how interlocking systems of power, such as cisheterosexism, racism, and economic exploitation, mutually shape one another, producing unequal material conditions and distinctive social experiences. This integration foregrounds PST as politically mediated and materially structured (Collins, 2020; Singh et al., 2023). In doing so, we aimed to expand existing trauma paradigms by highlighting the structural effects of cisheteronormativity and colonial violence in Latin America, while centering the potential of activism as a practice through which PST may be addressed and its systemic, material, and institutional origins confronted.

Engagement in 2SLGBTQIA+ activism has been reported to generate both positive and negative effects, depending on population, context, and the intensity of conflict exposure (Pepin‐Neff & Wynter, 2020; Szymanski et al., 2023). Activism has been associated with a heightened sense of life purpose, stronger community connectedness, and heightened critical consciousness, all of which are linked to improved well‐being (Frost et al., 2019; Obenauf et al., 2024; Paceley et al., 2021). Conversely, activism can be accompanied by significant burdens, such as retraumatization, emotional exhaustion, and burnout (Pepin‐Neff & Wynter, 2020). In one study, Pepin‐Neff and Wynter (2020) found that activism fostered pride, purpose, and identity affirmation; however, the findings also suggested that participation in activism was associated with considerable stress, including burnout, a fear of being “outed,” and exclusion, particularly for trans, intersex, and racialized activists. In another study, Conner et al. (2023) found that queer youth activists in the United States experienced higher levels of emotional labor than their cisgender heterosexual peers, with many participants describing activism as both necessary for survival and a source of psychological burden.

In Brazil, the limited research with 2SLGBTQIA+ populations has mostly focused on experiences of violence and its damaging effects (De Tilio & Gomes Silveira, 2021; Oliveira & Pontes, 2021). Existing scholarship suggests that these populations experience higher rates of anxiety, depression, suicidal behavior, and posttraumatic stress disorder (PTSD) symptoms than their cisgender heterosexual counterparts (Carneiro et al., 2024; Torres et al., 2021). Disparities are also evident in alcohol consumption, smoking, COVID‐19 infection, and barriers to health care (Jesus et al., 2020; Torres et al., 2021). This literature has predominantly been organized around a deficit‐focused orientation, emphasizing harm and risk while paying less attention to agency and resistance. Research addressing PST in the context of 2SLGBTQIA+ activism remains even more scarce and largely reproduces this analytic orientation, centering on the negative effects of political exposure and vicarious traumatization (De Tilio & Gomes Silveira, 2021; Oliveira & Pontes, 2021). Taken together, these limitations underscore the need to examine PST and resistance in relation to the historical conditions through which power relations and political mobilization have shaped 2SLGBTQIA+ trajectories in Brazil.

The struggles of 2SLGBTQIA+ populations in Brazil must be situated within a longer history of colonial and religious violence that has sought to eradicate sexual and gender diversity (Mott, 2015). Before the European invasion, which began around 1500, Indigenous peoples embodied plural configurations of what are today defined as SOGIE. Sixteenth‐century archives not only attest to these forms of fluidity but also document centuries of systematic persecution, torture, and executions of sexual and gender dissidents by the Portuguese Catholic Inquisition. This historical violence entrenched cisheteronormativity in Brazil's dominant culture and continues to perpetuate “transgenerational trauma” (Martín‐Baró, 1989) through institutions such as the state, religion, education, and the patriarchy.

Contemporary 2SLGBTQIA+ struggles in Brazil unfold in a national context shaped by the enduring influence of Christianity. Approximately 84% of the Brazilian population identifies as Christian, with evangelical denominations emerging as the fastest‐growing and gaining strong representation in the political arena (Quinalha, 2022). The Evangelical Parliamentary Front, now one of the largest cross‐party coalitions in Brazil's National Congress, comprises over 40% of federal deputies and advances a religious fundamentalist agenda. This political–religious project seeks to control society by imposing a singular religious truth and patriarchal morality on public institutions, sustaining social control and entrenched hierarchies. These dynamics intensify the everyday conditions of PST for 2SLGBTQIA+ communities, as activists confront both direct attacks from religious groups and the broader institutionalization of exclusionary policies.

Despite significant legal advances, including the recognition of same‐sex marriage in 2011 and the criminalization of homophobia and transphobia in 2019—both achieved through judicial rather than legislative routes—these rights remain fragile and unevenly implemented (Jesus et al., 2020). Internationally, Brazil is often portrayed through the lens of its major metropolitan centers, where public representations of queer life emphasize sexual openness and visibility. Rio de Janeiro is frequently associated with eroticized images of Carnival, while São Paulo hosts the world's largest Pride parade, both of which project a narrative of progressive inclusion.

Yet, such portrayals obscure the realities faced by 2SLGBTQIA+ activists in midsized municipalities, where smaller populations produce heightened forced visibility and intensify exposure to community surveillance and control. In these contexts, conservative political and religious forces are reinforced by their ability to monitor and stigmatize gender and sexual dissidents. Local political trajectories are, therefore, deeply entangled with racial and class inequalities, uneven policy implementation, and intensified forms of social control that shape the conditions under which PST is structurally produced.

## METHOD

### Participants

Guided by a qualitative design and a participatory research approach (Borda, 2008), we collaborated with 10 2SLGBTQIA+ activists who had resided in São José dos Campos (SJC), a mid‐sized city in Brazil, for at least 5 years, engaging them as contributors across multiple stages of the study. Participants ranged in age from 25 to 63 years, and most reported current or past leadership roles in grassroots organizations. The sample included 60% trans and gender diverse (TGD) individuals and 40% gay individuals. Regarding SOGIE, three participants identified as both trans women and *travestis* (i.e., a feminine gender category widely used in South America, historically articulated outside biomedical frameworks and shaped by activist reappropriation), one as a trans woman, one as nonbinary, one as a trans man, one as a lesbian, and four as gay; categories were self‐described and not mutually exclusive. Regarding race, six participants self‐identified as White, three as Black, and one as Indigenous.

### Procedure

After a 6‐month process of trust‐building with activists, which included participating in advocacy initiatives, marches, and meetings with state representatives, the first author invited 15 activists representing key grassroots movements in SJC to collaborate on the study. Initial meetings with participants focused on determining how this collaboration could be meaningful to them. Ten activists decided to join the study and completed an informed consent form.

Activists framed the study as a form of historical documentation and emphasized that its outcomes should be made accessible to 2SLGBTQIA+ communities. They shaped the early phases of the project by refining its scope and objectives, identifying potential community benefits, such as a book recounting the history of movements in SJC, which is currently in progress. Based on these discussions, the interview protocol was collaboratively developed to capture participants’ activist trajectories, experiences with and interpretations of violence, and whether processes of resistance and conscientization were elaborated. The questions were deliberately open‐ended to allow participants to narrate their stories in their own terms, while also inviting reflection on both positive and negative aspects of activism, participation in collective spaces, and everyday practices of resistance and care. For example, participants were asked, “Can you talk about positive and negative experiences related to your participation in the movement?” All interviews were conducted in person by the first author, lasted an average of 90 min, and were audio‐recorded. Participants were asked to choose interview locations where they felt privacy and comfort.

All procedures carried out in this study followed the ethical guidelines established by the Brazilian National Research Ethics Commission (CONEP) and were approved by the Ethics Committee of the University of São Paulo in December 2023. The first author is a queer activist from Brazil who identifies as nonbinary; the second author is an activist from Colombia who identifies as a queer woman; and the third author, who is from Brazil and identifies as a cisgender man, is an LP scholar and a cofounder of an observatory on political violence. These diverse positionalities, situated across activism and academia, fostered reflexive dialogue and enriched our interpretative engagement with the data. They also brought ethical complexities to our interactions with participants, particularly given the contrasting material conditions between the authors and some of the collaborating activists, many of whom, especially TGD participants, faced economic precarity. To support equitable participation, we approached each encounter with care and sensitivity, providing practical assistance and shared meals whenever possible to nurture a sense of reciprocity and collective care.

Reflexivity was central to maintaining methodological and ethical rigor throughout the study. The authors continuously revisited assumptions arising from the contrast between their critical sociopolitical perspectives and the activists’ struggles for inclusion and full citizenship, often articulated through their pursuit of a legal framework. Intersectionality guided these reflections, ensuring that analyses and interactions considered the entanglement of systems of oppression. These reflexive exchanges, together with the use of field diaries, integrated multiple viewpoints and sources of insight, serving as a form of methodological triangulation.

### Data analysis

Our dataset consisted of field diaries and transcripts of semistructured interviews. The first author facilitated activist engagement, kept field diaries during advocacy activities, and conducted the interviews, and the second author transcribed the recordings. Both authors independently engaged with the dataset through an iterative, reflexive process of reading, coding, and developing themes. The third author participated in theme development and its validation. All authors engaged in reflexive dialogue, supported by analytic memos documenting interpretive decisions and shifts in analytic focus, to support analytic rigor.

We analyzed the material using reflexive thematic analysis (RTA; Braun & Clarke, 2012), which emphasizes the interpretative role of the researcher and the coconstruction of meaning through engagement with participants’ situated accounts. Our analysis was guided by a meta‐theoretical framework informed by LP, intersectionality, and dialectical thinking. Within this orientation, the analysis combined both deductive and inductive coding, whereby codes functioned as concise labels for features of the data that were potentially relevant to the research question. Deductive coding involved interpreting the data in dialogue with LP and intersectionality, including concepts such as psychosocial harm and trauma and conscientization (Martín‐Baró, 1989), whereas the inductive orientation remained grounded in close engagement with participants’ situated accounts. Through the clustering and refinement of codes, we generated themes as analytically constructed patterns of shared meaning that captured broader issues relevant to the research question: How are PST and resistance dialectically produced, lived, and addressed within 2SLGBTQIA+ activism?

Our analysis probed beneath the surface of activists’ accounts, attending to meanings that were not always immediately explicit but emerged through reflexive interpretation in dialogue with participants. For example, we interpreted processes of conscientization within activists’ ongoing, collective efforts to expose and contest intersecting systems of power that marginalize 2SLGBTQIA+ people, and these interpretations informed how we articulated and refined themes. All authors collaboratively reviewed provisional themes, and the first author discussed them with activists during coding and theme development. Analytic dialogue with activists further informed the refinement of codes and themes, grounding rigor in sustained engagement and theoretically informed interpretation. This combination of RTA with participatory, critical–interpretive processes foregrounded how meanings were coconstructed as situated and relational.

## RESULTS

The analytic process ended with the creation of four themes that highlight the dialectical interplay between PST and resistance mechanisms: (a) addressing psychosocial harm and trauma in the realm of sexual orientation and gender identity, (b) decoding and confronting intersecting power structures, (c) reclaiming and preserving collective memory, and (d) cultivating citizenship and political consciousness. Here, we elaborate on these themes with excerpts that ground the analysis in participants’ narratives.

### Addressing psychosocial harm and trauma in the realm of sexual orientation and gender identity

Most participants reported that their 2SLGBTQIA+ activism was oriented toward dismantling the structures that produce oppression. They relayed that this commitment often demanded intense time investment and placed them in situations of confrontation, including demonstrations and extenuating negotiations with government agents. At the same time, participants reported carrying the cumulative effects of sexuality‐ and gender‐based oppression in their personal lives, rooted in historical marginalization and reinforced by religious fundamentalism. They described traumatizing experiences as relational ruptures, homelessness, physical and psychological violence, and periods of engaging in sex work for survival. Participants stated that activism itself also produced additional burdens, such as exposure to political intimidation, the strain of unrecognized labor, and experiences of exhaustion. Still, these experiences were often approached in a dialectical fashion, as a participant explained, “I sacrificed a lot… I actually gave up my personal life, my health, because of activism. Over these 30 years, it has really consumed me. But at the same time, it also helped shape who I am.”

This narrative captures a sentiment shared by many participants. The same participant described suffering a stroke, which she associated with the accumulated effects of gender violence, survival sex work, family expulsion, and years of intense activism. Yet, she reflected that activism helped her see that her hardships stemmed from discrimination and exclusion for being trans. Family rejection, economic precarity, loneliness, and social humiliation related to SOGIE were frequently narrated as sources of trauma in activists’ personal lives.

Across trajectories, activism appeared simultaneously as a source of identity and purpose and as a site of exhaustion and embodied strain. Another participant reflected on how engagement in activism exposed her to forms of traumatization she had not encountered in her personal life: “I experienced far more trauma within activism than in my life with my family. The consequences of being an activist were far more cruel: I received death threats, had a cross shoved in my face, was yelled at and humiliated.” She emphasized that religious fundamentalism was particularly present in this violence.

In another account, a participant recalled how her evangelical family, drawing on religious fundamentalist beliefs, would repeatedly reprimand her gender‐nonconforming expression and subject her to coercive practices, including gathering around her at night, against her will, to pray for “demons to be cast out of her” so that she could “become a normal boy.” Despite such attacks, both participants framed activism as inseparable from their sense of purpose. As one expressed, “Joining activism was an awakening experience, I learned I was not alone.” These accounts illustrate conscientization in practice, as personal trauma is reinterpreted through collective struggle and transformed into renewed commitment to social justice.

Other participants described the strain that activism placed on their intimate relationships and personal lives. For some, it led to separations and emotional distancing. One activist attributed the end of a decade‐long romantic relationship to her prolonged absence and the temporal demands of her activist work. Another shared that her family of origin condemned her participation in activism, criticizing the visibility it brought as she gained local prominence. Yet, neither expressed regret. Both individuals emphasized that activism allowed them to cultivate chosen families and build networks of mutual support, political belonging, and collective identity that became profound sources of care and protection.

Amid the religious hostility, long hours of activism, and pressures of intense public exposure, participants found ways to cope with the psychosocial toll. Chosen families and the camaraderie of activist communities were often described as vital, offering solidarity, companionship, and everyday care. Some participants also turned to personal practices, such as meditation, prayer, or celebrating achievements, while others found support in therapy groups or their queer‐affirming activist spaces. Several activists highlighted participation in Afro–Brazilian spiritual practices as a counterpoint to exclusionary Christian fundamentalism, providing spaces of healing and communal connection. In this way, activism was interpreted as both a site of exposure and a form of resistance, where trauma was at times reexperienced but also resignified through collective struggle and conscientization.

### Decoding and confronting intersecting power structures

We interpreted decoding power structures as the gradual and collective processes through which participants cultivated political awareness and learned to identify oppression as systemic, thereby identifying and addressing the roots of PST. In their accounts, this involved naming the ways power was woven into everyday life, within families, intimate relationships, schools, workplaces, and health systems, while also situating these experiences in relation to broader histories of marginalization that produce PST. Participants often described this process as emerging through experiences of rejection and exclusion that became resignified as part of a wider political awakening, reorienting previously individualized suffering into a collective and political understanding of traumatizing experiences.

Some participants had engaged in social movements for decades and, through this accumulated experience, emphasized the need for a unified platform capable of bringing together diverse actors and agendas. This was reflected in the creation of the LGBTQIA+ Fórum of SJC, a coalition that unites a wide range of social and institutional forces, including 2SLGBTQIA+, Black, Indigenous, and feminist movements; the Regional Council of Social Workers; and the local chapter of the Brazilian Bar Association.

One long‐standing activist emphasized that the Fórum is essential for sustaining the progressive agenda of local movements over the long term, particularly in a context marked by economic precarity and governmental hostility. She noted that there is no financial support for activism in the city and that activists often draw on their own limited means: “On top of that, many of us have complicated living situations, no fixed income. That's why a lot of grassroots movements don't last more than a few years.” The Fórum was, thus, narrated as both a practical response to economic precarity and an example of how activists decode the sociopolitical context, where the limits of scarcity are confronted with the possibilities of collective organization and resistance. It was also framed as creating the conditions for sustaining activism over time.

Members of the Fórum described education as a priority and identified it as a key arena where inequality is both reproduced and contested. These participants emphasized that formal education should serve as a vehicle for combating exclusion, a means to discern and navigate society, and a pathway toward improved socioeconomic conditions. Yet, for many participants, it was instead experienced as a site of violence and rejection, particularly for trans people. All trans participants reported frequent and intense experiences of violence during their time in the educational system, especially in primary and secondary school. This was described as a source of lasting trauma, as one participant explained, “Many of us are forced out of school. We are expelled, not officially expelled, but our permanence is made impossible…by the amount of violence we face from other students, and many times from teachers, too.”

By collectively decoding oppression—that is, critically analyzing institutional and intersecting mechanisms that perpetuate violence against 2SLGBTQIA+ people within the educational system—some participants developed a project to foster educational attainment for TGD people. Another participant involved in this initiative explained, “The project Transformatura [Transgraduation] creates opportunities for trans people to finish primary and secondary school, for free.” She added that the project includes classes, learning materials, transportation, meals, and a celebration for individuals who complete it. This example illustrates an intersectional praxis that addresses sexuality‐ and gender‐based violence alongside the economic precarity that contributes to school discontinuation.

In another narrative, a participant reflected on how engaging in activism, alongside learning from more experienced activists, allowed him to find his voice in political spaces and examine the systems that had silenced him for decades. He explained that activism enabled him to make sense of long‐standing silencing experienced as a Black gay man raised in a low‐income periphery. His account indicated that multiple positionalities, including race, income, and spatial marginalization, intersect to shape exclusion and invisibilization; we interpreted this as an implicit intersectional analysis of power across norms, institutions, and daily life.

This political awakening, together with the Fórum and school‐based projects, illustrates how participation in activism supports the collective identification, decoding, and interrogation of systems of power, confronting compounding oppressions, including cisheteronormativity, racism, and economic precarity. Through these spaces, participants exposed and challenged how power is organized across institutions and everyday life. Political education within social movements, cross‐generational learning, and intersectional collaboration were narrated as central practices to tracing violence, its sources, and its normalization.

### Reclaiming and preserving collective memory

The systematic erasure of 2SLGBTQIA+ people from both local and national history was a significant concern among participants and was interpreted as a source of PST, insofar as it reproduces historical silencing, social exclusion, and dehumanizing relations across generations. In response, participants have been organizing a range of cultural and educational activities aimed at reclaiming and preserving collective memory as a way of addressing PST at its historical and structural roots. These efforts include the production and documentation of historical counternarratives, as well as the radical transformation of local traditions to embrace sexual and gender diversity.

Brazil's precolonial harvest festivals honored nature and seasonal cycles. Following the Portuguese invasion, Catholic evangelization rescripted these celebrations, imposing cisheteronormative roles and symbols (Quinalha, 2022). A prominent example is the Junina party, June harvest festivities that blend Catholic, pagan, and Indigenous elements, within which 2SLGBTQIA+ people have long been marginalized or excluded. One participant described how they have been transforming the Junina party:

We are reclaiming our traditions! Our Junina party is a beautiful way to resignify a traditional Brazilian festival that's so central in our collective imagination. So often, we, LGBT folks, were left out of these festivities because they involve dancing, rituals, and symbols that are extremely binary.

Interrogating the historical invisibilization of 2SLGBTQIA+ people in local traditions, activists also decode oppression, confronting the erasure of their existence by recreating traditions. The same participant explained that activists now organize a Junina party at an “LGBT nightclub, a space we consider safe and inclusive for our community.” Another activist expanded on this topic, explaining that while traditional Junina parties center on a narrative regarding a cisheteronormative wedding, the activists’ version features a queer wedding, sometimes involving multiple partners, to embrace diversity. Through subversive transformations of cultural traditions, activists are turning these celebrations into spaces of inclusion, joy, and resistance.

When discussing the importance of documenting 2SLGBTQIA+ history, activists often highlighted their own intellectual and artistic production, as well as their collaborations with researchers, universities, intellectuals, and other artists. Many regarded participation in scientific studies as a meaningful documentary and reflexive practice. In reference to the present study, one participant explained, “It is important to me, knowing that history isn't erased, that it will be documented. We can only have a better future by understanding our past through our references.” In this sense, the objectives of this study, which participants helped to shape, align with broader demands for documenting and disseminating knowledge about 2SLGBTQIA+ trajectories and resistance. Through these documentary and knowledge‐producing practices, some participants emphasized that their participation in, collaboration on, and production of critical research were oriented toward confronting prejudice and discrimination.

For example, in a related reflection, one participant noted that she frequently collaborates with scholars, participating in conferences, classes, and studies concerning 2SLGBTQIA+ people. She emphasized: “These are opportunities to make ourselves visible, to share our side of the story… I think it truly contributes to those who are studying, and to the kind of professionals they are becoming.” Paradoxically, illustrating the institutional limits of such collaborations, a different participant recalled that in 2023, activists collaborated with SJC's Department of Social Support to the Citizen on a guide about gender and sexual diversity in the city. After extensive meetings and the approval of the final version, the activists were abruptly informed that the municipality would not publish it. The episode was experienced as another instance of institutional hostility, reinforcing feelings of disregard for activist labor without a genuine commitment to change.

Taken together, these accounts frame memory work as insurgent knowledge production: Activists do not merely document history—they also produce and recount it from their own perspectives. They craft counternarratives that unsettle cisheteronormative traditions; lead and participate in research; and generate art, archives, and community records of 2SLGBTQIA+ histories. In doing so, activists consolidate shared references, assert epistemic authority, and transform cultural and scholarly arenas into sites of pedagogy, belonging, and resistance.

### Cultivating citizenship and political consciousness

A recurring issue that participants raised was the ongoing struggle to secure full citizenship for 2SLGBTQIA+ people in SJC. Although advocacy for inclusive public policies remains a core strategy of local movements, activists also highlighted the need to address immediate and systemic gaps in the provision of public services, as well as the difficulties in navigating and accessing public services when those are available.

In response to failures, neglect, and rights violations by municipal authorities, activists mobilized to implement alternative forms of support and care, particularly in areas where the state had withdrawn or proven antagonistic. Among these, the Diversity Reference Center (DRC) was described as a key intervention, providing a range of services intended to improve the material and psychosocial conditions of 2SLGBTQIA+ people. In Brazil, a DRC is a multipurpose facility offering legal, psychological, and social assistance, as well as referrals to state services such as public defenders, child protection councils, and health care institutions. One activist recounted that, in the absence of municipal collaboration and in the face of institutional hostility, a coalition of grassroots movements independently launched a local DRC without relying on public funding or infrastructure.

Participants articulated the importance of several initiatives of the DRC, including a monthly “Clotherack Project,” which distributes free clothing and hygiene kits; psychological support groups; a monthly community picnic aimed at reclaiming public space and providing welcoming leisure; and the co‐organization of the city's annual 2SLGBTQIA+ march each December. In combination, these efforts were framed as addressing gaps in public services; providing material support; cultivating emotional well‐being and community solidarity; and exemplifying collective self‐organization, resistance, and care in the face of institutional neglect.

Another widely resonant sentiment among participants was reflected in the collective efforts to secure citizenship through the organization of the first Municipal Conference on LGBTQIA+ Rights in SJC in 2025. One participant noted that these municipal conferences function as essential entry points in Brazil's participatory policy‐making structure, laying the groundwork for state‐level debates and culminating in the National Conference. This activist further highlighted that guidelines issued in 2024 by the Ministry of Human Rights stated that “each municipal government should organize its own LGBTQIA+ municipal conference, and in cases where the municipality fails to do so, civil society organizations are authorized to convene the conference.” He emphasized that after two negative responses from the municipality, the LGBTQIA+ Fórum deliberated to organize it independently. This participant explained that the conference agenda distilled the Fórum's priorities: (a) combating violence against 2SLGBTQIA+ people, (b) advancing income generation and employment, (c) foregrounding intersectionality and international connections, and (d) engaging with Brazil's national policy landscape.

Participants were particularly concerned with the last priority, which addresses engagement in national politics to establish a robust legal framework for 2SLGBTQIA+ rights. Another participant emphasized that the absence of comprehensive legislation leaves 2SLGBTQIA+ communities exposed to institutional neglect and uneven protections. She noted that, while judicial rulings have granted rights such as marriage equality and the criminalization of homophobia and transphobia through their equivalence to racism, there is still no cohesive body of national laws specifically safeguarding 2SLGBTQIA+ rights. “Just as there is a statute for older adults and for children and adolescents,” the activist argued, “we also want a statute or legal framework we can call our own, something that protects us.”

In both the municipal conference and the creation of the DRC, participants noted persistent institutional hostility from local authorities, which they countered through autonomous, grassroots organizing. These efforts reflected a shared commitment to full citizenship, expressed through systems of care, visibility, and political participation. The interplay between political advocacy and community‐based service provision exemplified an engaged citizenship grounded in collective self‐determination and public accountability. Together, these actions revealed a dynamic process of critical conscientization, where resistance and policy engagement sustain the ongoing struggle to transform oppressive social structures.

## DISCUSSION

Our analysis explored the tensions and contradictions narrated by 2SLGBTQIA+ activists, who dialectically engage with PST and resistance in the context of a conservative municipal administration and under conditions of material and political precarity, in a midsized city in Brazil. Rather than offering a linear path to healing, the activist trajectories we documented reflect complex processes of a collective resignification of traumatic experiences, political resistance, and reparation. The narrative positions activism as a privileged site for promoting critical consciousness, where suffering is politicized and resistance is enacted as a collective project of decoding oppressive power relations and structures, reclaiming historical and cultural participation, and demanding full citizenship.

Previous research with 2SLGBTQIA+, Black, and Indigenous activists has shown that participation in organized collectives fosters the development of critical consciousness through shared reflection and the collective analysis of social positions (Matsumoto et al., 2024; Paceley et al., 2021). Such processes deepen understanding of the systems, conditions, and dynamics that sustain marginalization (Gonçalves et al., 2017). Our study corroborates these insights. Activists in SJC described how political conscientization oriented their actions toward prioritizing the most oppressed individuals, particularly low‐income TGD people, who often face disproportionately poor health and psychosocial outcomes (Carneiro et al., 2024). For example, activists in SJC sought to fill institutional voids by providing urgently needed community‐based services, ranging from gender‐affirming spaces to psychological support groups, grounding their practices in the politics of collective care, as seen in similar studies in the United States (Frost et al., 2019; Sostre et al., 2024).

Across the coconstructed themes, we interpreted a broader phenomenon of collective resignification of traumatic experiences, through which activists elaborated on psychosocial harm in ways that fostered political clarity and collective agency. Participants recounted recurrent experiences of trauma linked to violence at school; expulsion from their family homes; exclusion from the formal labor market, which often led to survival sex work (particularly among TGD people); and persecution driven by religious fundamentalism. These narratives indicated that activism became a space to work through psychosocial suffering by situating it within shared histories of oppression and unveiling the systems that sustain marginalization. This interpretation reflects a latent pattern in how traumatic experiences were continuously narrated and reframed through collective praxis, echoing findings among Black TGD activists in the United States, where collective action against gendered racism and cissexism was enacted as both a response to intersecting traumatic experiences and a means of cultivating visibility, solidarity, and community care within activism spaces (Sostre et al., 2024).

Through critical reflection, traumatizing experiences were resignified as collective memories of resistance that fuel social transformation, resonating with processes of conscientization observed among Indigenous movements in Brazil, where the documentation of collective trauma became a means to expose state violence and to demand historical reparation and territorial rights (Gonçalves, 2017). For activists in SJC, reparation was not only demanded but enacted as a multisystemic process, involving the reconfiguration of power relations through legal advocacy, collective and safe occupation of public spaces, formation of chosen families, and recreation of traditions in SOGIE‐affirming ways. These practices illustrate how PST was addressed not through individual recovery but through collective action that bridges personal and structural transformation, laying the groundwork for the networks of solidarity and coalition‐building that sustain community resistance.

Community resistance in SJC was deeply intertwined with coalition‐building and intersectional solidarity across social movements. Activists frequently described their political resistance as a collective endeavor sustained through intersectional alliances. Three major coalition initiatives exemplified how independent activists and grassroots collectives have been organizing from a shared human rights perspective: the LGBTQIA+ Fórum of SJC, the DRC, and the Municipal Conference on LGBTQIA+ Rights. When recounting the history of local organizing, participants emphasized that coalitions were also essential for ensuring the continuity of advocacy efforts over the long term. Local social movements often had short life spans due to material precarity and the intense emotional and temporal demands of activism. Coalitions, as an aspect of community resistance, also contributed to greater visibility and legitimacy in public arenas, allowing activists to sustain their social justice agenda through a broader network of collaborators committed to advancing interconnected human rights causes.

We emphasize that the psychosocial approach to trauma, unlike individualistic models of psychological trauma, centers on sources of collective suffering rather than its symptoms. It highlights the structural, relational, and historical conditions that generate and reproduce psychosocial harm. From this perspective, PST stems from social and political processes that reflect dehumanizing relations and systemic violence. Within activism, this understanding is dialectical: Traumatic experiences and resistance are mutually shaping forces. Through processes of conscientization, activists reinterpret their suffering in relation to broader structures of oppression, cultivating political awareness that fosters collective action. In this dialectic, suffering becomes politicized, and resistance emerges as a possibility of supersession through the reinterpretation of traumatic experiences into shared purpose, care, and struggle for social justice.

The limitations of this study relate to both methodological and epistemological dimensions. Among the few activists in SJC with long trajectories of activism work, only 10 agreed to participate in the kind of participatory engagement this approach required, a group we considered sufficient to sustain theoretically rich theme development (Braun & Clarke, 2012). This judgement was grounded in the depth of dialogue and the iterative nature of our engagement. Alongside the interviews, advocacy work and postinterview dialogues further deepened the analysis. Nonetheless, the study could have benefited from broader integration of field diaries and observational materials, which would have expanded opportunities for methodological triangulation. Additionally, tensions emerged between our critical theoretical orientation and the perspectives prioritized by activists. Although our LP and intersectional lens anticipated more explicitly antineoliberal framings, participants often emphasized legal recognition and full citizenship, likely as a result of their intense coalitions with professional councils and human rights specialists. Remaining faithful to their voices required foregrounding these narratives, even when they diverged from our expectations. We interpret these contradictions as part of the dialectical complexity of conscientization, where strategies for survival and transformation arise within, against, or beyond liberal rights frameworks.

For health professionals and researchers in trauma‐oriented work, as well as those involved in activism, we recommend a liberation‐centered, intersectional, and community‐anchored practice that treats PST as the embodiment of dehumanizing relations, not only as the presence of individual symptoms. In practical terms, this means there is a need to codesign care with 2SLGBTQIA+ activists; facilitate peer‐led spaces for critical reflection and collective care; and embed support in grassroots venues that activists already sustain, such as the DRC and the LGBTQIA+ Fórum, while partnering with school initiatives that secure educational continuity for 2SLGBTQIA+ people. These practices stabilize distress, politicize pain, and share the load, advancing conscientization alongside material support. Professionals should act as collaborators rather than gatekeepers; provide reflective supervision to mitigate vicarious traumatization; find ways to compensate activist time; and measure success by strengthened collective agency, reduced isolation, and safer participation in public life, rather than by symptom reduction alone. Finally, future collaborations should systematically integrate field diaries and observational materials, expanding triangulation and deepening interpretation without recentralizing professional authority.

We propose that the central dynamic of 2SLGBTQIA+ activism in Brazil lies in the cultivation of critical consciousness: a dialectical process through which PST and collective resistance are continuously negotiated. Activism often exposes participants to conflict, exhaustion, and emotional strain, yet it also enables the politicization of pain, reclamation of historical memory, and reorientation of suffering into shared purpose and agency. Our analysis underscores that confronting PST cannot be separated from the collective processes of conscientization that sustain community‐building, solidarity, and social transformation. We recommend that social movements, health professionals, and policymakers actively support spaces that nurture these reflexive and politicizing practices, while fostering conditions for self and collective care among activists.

## AUTHOR NOTE

We have no conflicts of interest to disclose. We thank the Coordination for the Improvement of Higher Education Personnel (CAPES) Brazil, the University of Groningen, and the research project “K‐Reporters, Reassembling politics across children's cultures to scale intersectional pedagogies (Horizon Europe MSCA Staff Exchange; Ref. 101131090)” for funding this study.

## OPEN PRACTICES STATEMENT

For ethical reasons, the full dataset cannot be made publicly available. The public sharing of complete narratives was not stipulated in the informed consent process, and participants shared sensitive information related to their health and psychosocial experiences. In line with standard qualitative research practices, we provide a minimal relevant dataset through illustrative quotes supporting the thematic analysis.

## AUTHOR CONTRIBUTIONS

**Gab C. Siqueira**: conceptualization, investigation, funding acquisition, methodology, data curation, writing–original draft, formal analysis, project administration. **Luisa F. Velásquez Vallejo**: validation, writing–original draft, formal analysis, data curation, writing–review and editing, visualization. **Antonio Euzebios Filho**: conceptualization, methodology, validation, writing–review and editing, supervision.
